# High efficiency all-polymer tandem solar cells

**DOI:** 10.1038/srep26459

**Published:** 2016-05-26

**Authors:** Jianyu Yuan, Jinan Gu, Guozheng Shi, Jianxia Sun, Hai-Qiao Wang, Wanli Ma

**Affiliations:** 1 Institute of Functional Nano & Soft Materials (FUNSOM), Jiangsu Key Laboratory for Carbon-Based Functional Materials & Devices, Soochow University, 199 Ren’ai Road, Suzhou, 215123, Jiangsu, PR China

## Abstract

In this work, we have reported for the first time an efficient all-polymer tandem cell using identical sub-cells based on P2F-DO:N2200. A high power conversion efficiency (PCE) of 6.70% was achieved, which is among the highest efficiencies for all polymer solar cells and 43% larger than the PCE of single junction cell. The largely improved device performance can be mainly attributed to the enhanced absorption of tandem cell. Meanwhile, the carrier collection in device remains efficient by optimizing the recombination layer and sub-cell film thickness. Thus tandem structure can become an easy approach to effectively boost the performance of current all polymer solar cells.

Polymer solar cells (PSCs) have attracted a great deal of attention in recent years. Not only are polymer solar cells lightweight and flexible, they are transparent in the visible range, inexpensive and easy to manufacture. Additionally, there exists a wide range of available materials for use as the active materials in these photovoltaic devices^1^,^2^,^3^,^4^,^5^. The PCEs of polymer solar cells have increased rapidly in the past few years as a result of improved materials and device architecture. Recently, Hsiang *et al.* achieved a PCE over 8.5% by using Porphyrin-incorporated 2D D/A polymers in organic solar cells^6^. Wei *et al.* employed two donors in the ternary blend to form an alloy and obtained a PCE of 10.5%^7^. Ade and Yan have achieved 10.8% PCE for polymer/fullerene composites after careful optimization^8^. Fullerenes, with excellent electron mobility, are usually used as the acceptor materials in polymer solar cells. Although fullerene derivatives have many advantages, conventional PC_61_BM (phenyl-C61-butyric acid methyl ester) demonstrates weak absorbance in the visible region, resulting in inefficient photon harvest. In addition, fullerenes tend to aggregate under elevated temperatures, causing deteriorated morphology and consequently reduced lifetime of PSCs^9^,^10^,^11^.

Using polymers as both electron donors and acceptors is considered as an effective strategy to solve these problems. Compared with polymer/fullerene system, all-polymer solar cells (all-PSCs) demonstrate many superior properties, such as enhanced optical absorption and mechanical properties, as well as greater synthetic flexibility in tuning the acceptor material properties^12^,^13^. Rapid developments in all-polymer solar cell technology have taken place in the last two years. Much progress in boosting the efficiency of polymer/polymer solar cells has been demonstrated. Hwang *et al.* designed a series of new semiconducting naphthalene diimide (NDI)-selenophene/perylene diimide (PDI)-selenophene random copolymers, and achieved a PCE of 6.3% by optimizing the proportion of PDI in copolymers^14^. Jung *et al.* employed Fluoro-substituted n-type conjugated polymers to achieve a PCE of 6.71% without using any additives^15^. The PCE of 7.7% for single junction all-polymer cells was achieved by Hwang and his coworkers by controlling the self-organization rate of the polymer blend film^16^. And very recently, new high PCE of 8.27% was reported for all-polymer solar cells by Li *et al.*^17^. Despite its early promise, the PCE of the all-polymer solar cell still lags behind that of polymer-fullerene solar cell. More strategies should be adopted to further increase the PCE of all-polymer solar cells.

Tandem structure has been widely reported for enhancing the PCE in organic solar cells as a result of better utilization of the solar spectrum^18^,^19^,^20^,^21^,^22^,^23^,^24^,^25^,^26^,^27^,^28^. By using polymers with complementary absorption, tandem cell can broaden the device absorption to near infrared region, covering more solar spectrum. Meanwhile, the potential loss during photo-to-electron conversion process can be reduced by using tandem structure. To date, the highest PCE of 11.3% has been reported for polymer tandem solar cell using sub-cells with different absorption^29^. However, this structure requires the two sub-cells are both efficient and with complementary absorbance. On the other hand, the optimal thickness of active layers in PSCs (especially in all-PSCs) is usually around 100 nm since the carrier mobility of organic materials is relatively low, which thus limits the overall film absorption between 60% to 80%^30^,^31^,^32^,^33^,^34^,^35^,^36^,^37^. Therefore using tandem structure with the same sub-cells can potentially further enhance the absorption and hence improve the device PCE. This strategy requires only one efficient D/A composite and the processing conditions are relatively simpler. Indeed, a few organic tandem solar cells with identical sub-cells have been reported previously^28^,^38^,^39^,^40^. However, it is worth noting that no tandem structure has been reported in all-PSCs till now, likely due to the difficulty to select two D/A composites with high efficiency and complementary absorbance simultaneously. Thus the strategy to use identical sub-cells in fabricating tandem devices is especially useful for all-PSCs. In this work, we reported an efficient all polymer tandem solar cell for the first time. Polymer P2F-DO with a broad absorption from 400–800 nm was used as the donor material. The P2F-DO:NDI2OD-T2(N2200) system shows high performance in single junction solar cells. Tandem cells based on P2F-DO:NDI2OD-T2(N2200) system were fabricated, with inverted structures to achieve better device stability. As a result, improved power conversion efficiency of 6.70% was achieved for the tandem device, which is over 43% higher than the 4.68% PCE of the optimized single cell. This work demonstrates the promising potential to employ tandem structure in all-polymer solar cells.

## Results

**Figure 1:**
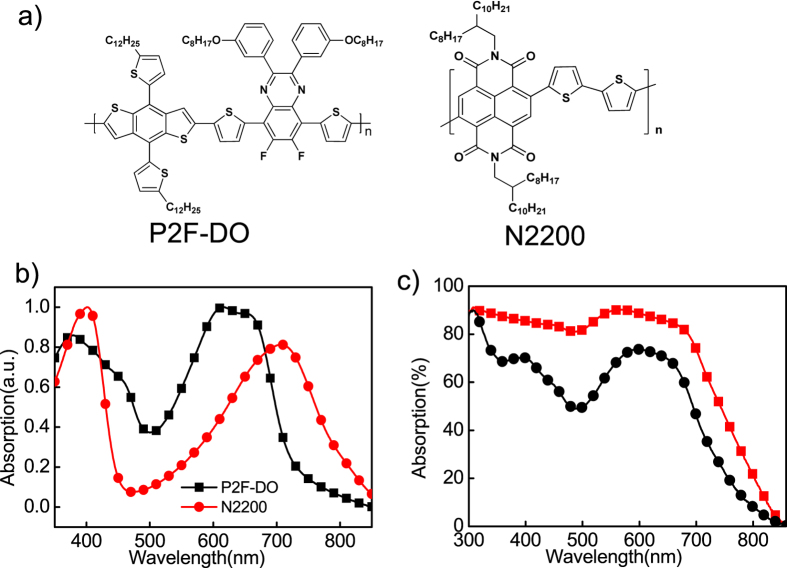
(**a**) Chemical structures of polymer (P2F-DO) and polymer (N2200). (**b**) Normalized ultraviolet-visible absorption spectra of P2F-DO and N2200. (**c**) The total absorption of the P2F-DO:N2200 blend film in a tandem cell (red) and single cell (black) device.

Chemical structures of the polymer donor P2F-DO and the polymer acceptor N2200 are shown in Fig. 1a. The synthesis method of P2F-DO is reported elsewhere^41^. Figure 1b shows normalized ultraviolet-visible absorption spectra of the two polymers. Both polymers show broad absorption from 300 nm to over 800 nm and their absorption spectra are partially complementary. The total film absorption of the P2F-DO:N2200 blend layers is shown in Fig. 1c by using a integrating sphere. We can see that the blend film absorption for tandem cells is significantly higher than that for single cell. Thus the use of tandem structure can largely improve the device absorption and meanwhile avoid the carrier recombination resulting from thick film.

**Figure 2:**
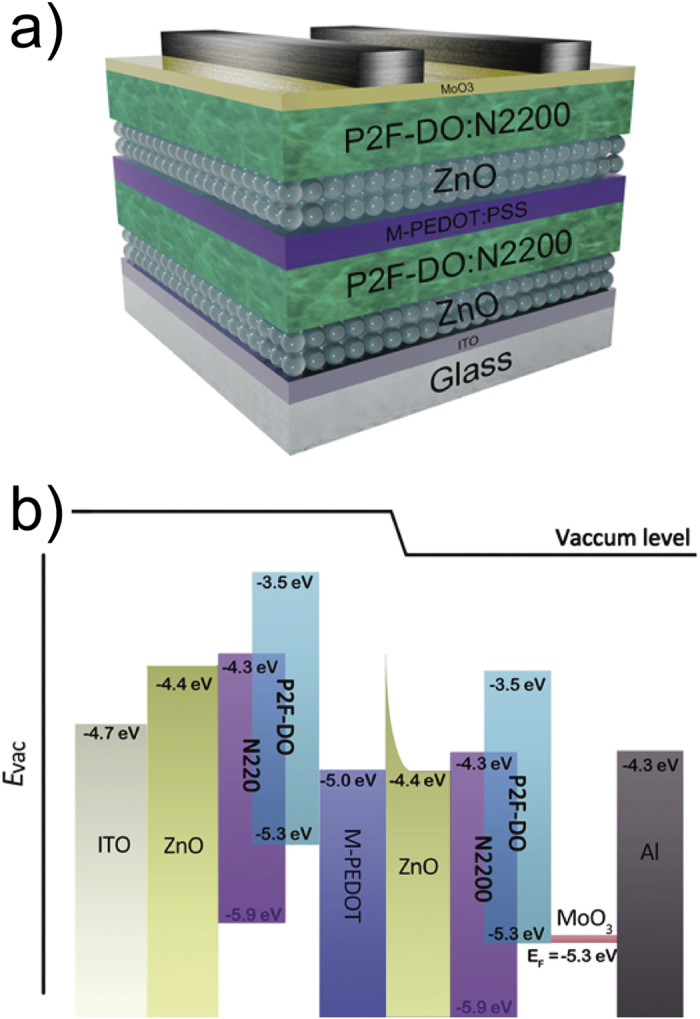
(**a**) Tandem device structure: glass/ITO/ZnO/P2F-DO:N2200/M-PEDOT:PSS/ZnO/P2F-DO:N2200/MoO_3_/Al. (**b**) Energy levels of materials used in tandem devices.

The tandem device structure and energy levels for the used materials are shown in Fig. 2a,b, respectively. Inverted device structure with zinc oxide (ZnO) was used to enhance the device stability^42^. A simple interconnecting layer of M-PEDOT:PSS/ZnO was adopted to connect the two sub-cells. The recombination layer plays an important role in tandem devices, which requires a resistance-free electrical connection to minimize electric potential loss between sub cells. And it must be transparent enough to minimize absorption losses. In addition, the recombination layer should be the recombination zone to align the quasi-Fermi level of both holes and electrons, which enables a more efficient recombination. More importantly, to fabricate a successful tandem device, the recombination layer should be physically robust to protect the underlying layers from the solution process of the upper layers^43^. The combination of M-PEDOT:PSS and ZnO have been confirmed to be an efficient recombination layer in previous works^23^,^26^. The M-PEDOT:PSS works as the hole transport layer and ZnO as the electron transport layer. In our work, the optimal thickness of the recombination layers is about 90 nm, with a 50 nm M-PEDOT:PSS and a 40 nm ZnO layer. The film thickness is small enough to ensure the transmissivity of the light. Meanwhile, the combination layer shows good electrical connection in these tandem devices, and the layers also serve well as the protection layer, strong enough to avoid the penetration of the solvent during the coating of upper layer in our experiment. For each processing condition, six devices were fabricated to assure the data reproducibility. The details of ZnO synthesis and device fabrication as well as characterizations are provided in the experiment section.

**Figure 3:**
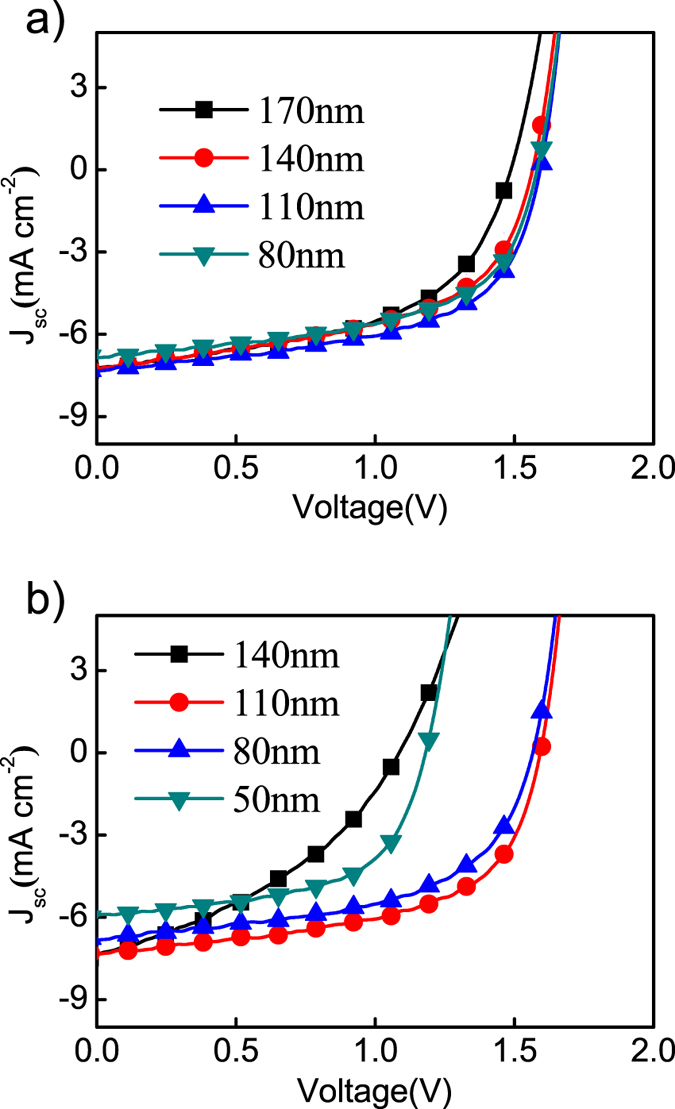
(**a**) J-V curves of the tandem devices with different thickness of bottom cell (The thickness of top cell is fixed at 110 nm). (**b**) J-V curves of the tandem devices with different thickness of top cell (the thickness of bottom cell is fixed at 110 nm).

At first, single junction solar cells were fabricated with an inverted structure of ITO/ZnO/P2F-DO:N2200/MoO_3_/Al. The photovoltaic parameters are summarized in Table 1. An optimal PCE of 4.68% was obtained with an open circuit voltage (Voc) of 0.8 V, a Jsc of 10.08 mA cm^−2^ and a fill factor (FF) of 58%. The thickness of the active layer for the optimized single cell is around 110 nm (See Table S1). We investigated the non-geminate recombination of devices with different film thickness by adjusting the incident light intensity^44^. The results are shown in Figure S2. The tandem solar cells were then fabricated with the configuration of ITO//ZnO/P2F-DO:N2200/M-PEDOT:PSS/ZnO/P2F-DO:N2200/MoO_3_/Al. It is well-known that the thickness of the sub-cell is the critical factor for the PCEs of the tandem devices^28^. The current balance between the top and bottom sub-cells can be achieved by finely tuning the thickness of each sub-cell, since the current is largely determined by the film absorption^45^. We thus fixed the thickness of the bottom or the top cell and then adjust the thickness of the other sub cell until the optimal device performance was obtained. The fixed thickness for the sub-cell is 110 nm, since excellent absorption and charge collection can be achieved simultaneously at this thickness. The experiment results are shown in Fig. 3, with the detailed parameters listed in Table 2. In Fig. 3a, the top cell thickness is fixed at 110 nm while the thickness of bottom cell is varied from 80 nm to 170 nm to reach the current balance. Judging from the J-V curves and data in Table 2, the best device performance is achieved for the tandem device with the thickness of bottom cell at 110 nm. The highest PCE is 6.70%, with a Jsc of 7.31 mA cm^−2^, a Voc of 1.58 V, and a FF of 58%, which is significantly higher than the PCE of single cell. Any deviation from 110 nm for the thickness of active layer results in decreased device performance. For example, for a thick film of 170 nm, the FF and Voc are decreased, both indicating increased charge recombination which is likely caused by the large film thickness. In contrast, for a thin film of 80 nm, the Jsc is significantly decreased which can be attributed to the weak absorption of the bottom layer. These results show the strong correlation between the PCE and the thickness of the sub-cell film. The effect of the top-cell thickness on the device performance was also investigated, as shown in Fig. 3b. In this investigation, the active-layer thickness of the bottom cell is fixed at 110 nm while the thickness of the top-cell thickness varies from 50 nm to 140 nm. 110 nm is also found to be the optimal thickness for the top cell. If the thickness of the active layer in the top cell increases above the optimal 110 nm, FF and Voc drop significantly as a result of large serial resistance. If the thickness is smaller than 110 nm, we observe the device Jsc is dramatically decreased as a result of reduced absorption. Interestingly, the Voc is also decreased, likely due to unbalanced current between the sub-cells. In short, the performance of all-PSC tandem cells is very sensitive to the sub-cell thickness. Our experiments reveal that the optimal PCE is achieved when the two sub-cells have the same film thickness of 110 nm.

**Figure 4:**
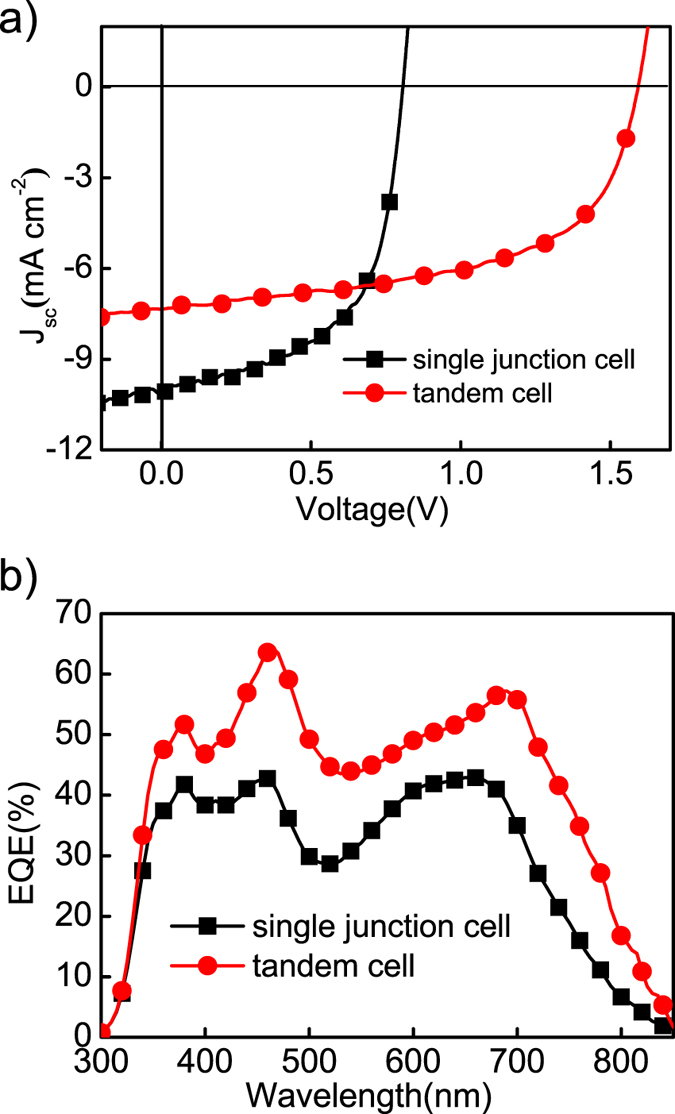
(**a**) *J-V* curves of both single junction and tandem cells. (**b**) Measured external quantum efficiency of single junction solar cells and calculated EQE of the tandem solar cells.

The J-V curves of the optimized single junction and tandem cells are shown in Fig. 4a, with the corresponding photovoltaic parameters listed in Table 1. We can see that the Voc of the tandem cell is equal to the sum of two sub-cells’ and the FF is the same for both devices, indicating minimally increased recombination after stacking of the two sub-cells. After film thickness optimization, the Jsc of tandem device is 7.31 mA/cm^−2^, which is about 73% of the optimal single-cell current 10.08 mA/cm^−2^. Since the Jsc of tandem cell is limited by the sub-cell with the smaller current, the total current generated by the two sub-cells is thus larger than 14.62 mA/cm^−2^, which is apparently higher than that of the single cell. We can then conclude that our optimized all-polymer single cell cannot fully utilize the incident photons, since the film thickness has to be thin to achieve efficient charge extraction. Tandem structure, with doubled film thickness, can thus achieve largely improved absorption without sacrificing the charge exaction efficiency, which is therefore a simple approach to boost the efficiency of the current all-PSCs. It is necessary to measure the external quantum efficiency (EQE) of tandem devices to confirm the high performance. However, EQE measurements with bias lights do not fit in such a structure since both sub-cells have identical light response. So, we measured the reflectivity of the devices and calculated the internal quantum efficiency (IQE) of the single junction cell^28^. Then we assume the sub cell has the same IQE. Thus the EQE of the tandem cell can be estimated, as shown in Fig. 4b. The Integrated current density of the single cell is 9.13 mA/cm^2^, close to the measured 10.08 mA/cm^−2^. We can also observe that the EQE of tandem cell is generally higher than that of single cell over all the absorption range, suggesting enhanced light harvesting by using tandem structure with identical sub-cells.

## Conclusion

In summary, we have reported for the first time an efficient all-polymer tandem cell using identical sub-cells based on P2F-DO:N2200. A high PCE of 6.70% was achieved, which is among the highest efficiencies for all polymer solar cells and 43% larger than the PCE of single junction cell. The largely improved device performance can be mainly attributed to the enhanced absorption of tandem cell. Meanwhile, the carrier collection in device remains efficient by optimizing the recombination layer and sub-cell film thickness. Thus tandem structure can become an easy approach to effectively boost the performance of current all polymer solar cells.

## Methods

### Synthesis of ZnO Nanoparticles

ZnO nanoparticles synthesis was performed with a modified recipe according to the paper^46^. 1.1 g (5 mmol) of zinc acetate dihydrate (ZnAc2•H2O) was dissolved in 76 ml MeOH in a three necked bottle, heating to 60 °C for 30 min. 0.57 g KOH was added to 24 ml MeOH, and this solution was added dropwise to ZnAc2•H2O solution under vigorous stirring. The mixed solution was stirred at 60 °C for 2 h. Once cooled, the solution was divided into three tubes and centrifuged at 5000 rpm for 5 min. The residual solution was removed and then 30 mL MeOH was added into each tube followed by vigorous vibration. The centrifugation step was repeated twice. The resulting dry nanoparticles was treated with CF, MeOH, n-butanol with a ratio of 1:1:8 to obtain a concentration of 8 mg/mL. Before use, the ZnO nanoparticle solution was filtered by a 0.22 mm PVDF syringe filter.

### Fabrication of single cell

The glass substrate coated with ITO was cleaned by sequential sonication in acetone, deionized water, isopropanol and acetone, and treated with UV-ozone for 20 min. ZnO nanoparticles were then spin-coated at 1500 rpm on the substrate and heated at 120 °C for 2 min. The last step was repeated and the sample was heated at 120 °C for 5 min. When it cooled down, the active layer (P2F-DO and N2200 with a mass ratio of 2:1 were dissolved in chloroform with a concentration of 10 mg/mL) were spin-coated at 3000 rpm to obtain a thickness of 110 nm, then heated at 100 °C for 1 min. Before spin-coating of the active layer, the solution should be kept heating at 50 °C for at least 2 h. After the spin-coating, thin layers of 8 nm MoO_3_ and 80 nm of Al were deposited via the methods of thermal evaporation. The area of each device is 0.0725 cm^−2^.

### Fabrication of tandem devices

The bottom cell was fabricated as the single junction cell produced. After that, M-PEDOT:PSS was spin-coated on the surface of the active layer at a speed of 1300 rpm^47^, then heated for 5 min at 120 °C, then the process is just like the fabrication of the bottom cell. The thickness of the active layer was adjusted by controlling the speed of spin-coating. Finally, thin layers of 8 nm MoO3 and 80 nm of Al were deposited by the methods of thermal evaporation. The area of each device is also 0.0725 cm^−2^.

### Device characterization

The current density–voltage characteristics of the solar cells were measured with the Keithley 2400 (I–V) digital source meter under a simulated AM 1.5 G solar irradiation of 100 mW cm^−2^ (Newport, Class AAA solar simulator, 94023A-U). The light intensity is calibrated by the certified Oriel Reference Cell (91150V) and verified with the NREL calibrated Hamamatsu S1787–04 diode. The external quantum efficiency (EQE) was recorded using a certified IPCE instrument (Zolix Instruments, Inc, Solar Cell Scan100). UV-vis-NIR spectra were recorded on a Perkin Elmer model Lambda 750.

## Additional Information

**How to cite this article**: Gu, J. *et al.* High efficiency all-polymer tandem solar cells. *Sci. Rep.* **6**, 26459; doi: 10.1038/srep26459 (2016).

## Supplementary Material

Supplementary Information

## Figures and Tables

**Table 1:** Device parameters of optimized single junction solar cell and tandem solar cell based on P2F-DO:N2200.

	J_sc_/mA/cm^2^	V_oc_/V	FF	PCE/%
Tandem cell	7.31	1.58	0.58	6.70
Single cell	10.08	0.80	0.58	4.68

**Table 2:** Device performance of tandem solar cells with different thickness of sub-cells.

Bottom thickness/nm	Top thickness/nm	J_sc_/mA/cm^2^	V_oc_/V	FF	PCE/%
170	110	7.20	1.49	0.53	5.69
140	110	7.20	1.55	0.54	6.03
110	110	7.31	1.58	0.58	6.70
80	110	6.82	1.58	0.57	6.14
110	140	7.26	1.08	0.39	3.06
110	80	6.80	1.55	0.56	5.90
110	50	5.89	1.17	0.59	4.07
