# Supplementary material for: High efficiency all-polymer tandem solar cells
Source: Sci Rep. 2016 May 26;6:26459. doi: 10.1038/srep26459 (PMC4881030; doi:10.1038/srep26459)
Supplement: Supplementary Information [file srep26459-s1.docx]

**Supporting information**

High efficiency all-polymer tandem solar cells

Jinan Gu^+^, Guozheng Shi^+^, Jianxia Sun, Hai-Qiao Wang and Wanli Ma*.

* wlma@suda.edu.cn

Institute of Functional Nano & Soft Materials (FUNSOM), Jiangsu Key Laboratory for Carbon-Based Functional Materials & Devices, Soochow University, 199 Ren'ai Road, Suzhou, 215123, Jiangsu, PR China


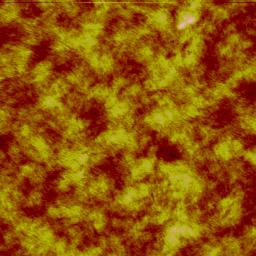

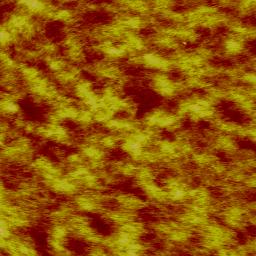

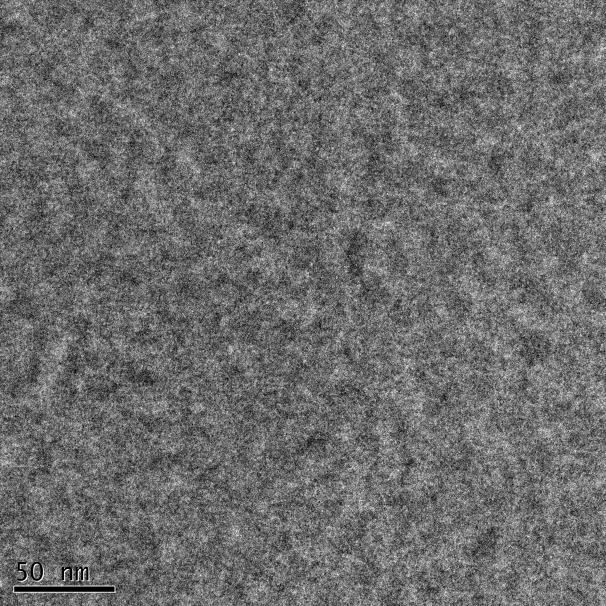

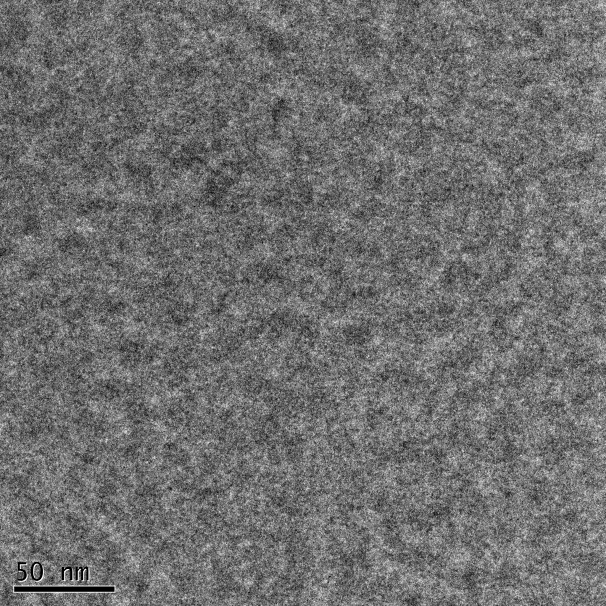


**15nm**


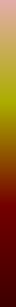


**15nm**


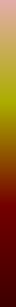


a)

b)

c)

d)

Figure S1. a) Atomic force microscopy (AFM) height image of as cast P2D-DO: N2200 blend films (2um*2um). b) film heated at 100 ^o^C for 1 minute. c) Transmission electron microscope (TEM) image of as cast P2D-DO: N2200 blend film. d) film heated at 100 ^o^C for 1 minute.

**Non-geminate Recombination Measurement**

Non-geminate Recombination is one of the important factors determining the device performance. Devices were fabricated with a structure of ITO/ZnO / P2D-DO: N2200/MoO_3_/Al. We studied the change of short circuit current density (Jsc) with incident light intensity to gain deeper insight into the charge recombination kinetics. The light intensity-dependent Jsc were measured under various light intensities from 100 to 1 mW/cm^2^. The relationship between Jsc and the intensity of the light can be represented using the power law equation:

Jsc $\propto$light intensity^α^ equation 1

Where α is indicative of efficient sweep-out of carriers prior to recombination. At short circuit, the bimolecular recombination should be minimum (α~1) for a maximum carrier sweep out, and any deviation from α~1 implies non-geminate recombination^44^

Figure. S2 The measured J_SC_ of single junction cell with different thickness plotted against the light intensity (symbol) on the logarithmic scale and the fitted power law (line) yield α.

Table. S1 Device performance and α of single junction solar cells with different thickness.

|  | J_sc_/mA/cm^2^ | V_oc_/V | FF | PCE/% | α |
| --- | --- | --- | --- | --- | --- |
| 80nm | 10.21 | 0.82 | 0.56 | 4.69 | 0.842 |
| 110nm | 10.65 | 0.82 | 0.55 | 4.80 | 0.838 |
| 140nm | 10.75 | 0.82 | 0.48 | 4.23 | 0.818 |
